# Why is there persistent disease despite biologic therapy? Importance of early intervention

**DOI:** 10.1186/ar4594

**Published:** 2014-06-26

**Authors:** Paul Emery

**Affiliations:** 1 Leeds Institute of Rheumatic and Musculoskeletal Medicine, University of Leeds, Chapel Allerton Hospital, Chapeltown Road, Leeds LS7 4SA, UK; 2 NIHR Leeds Musculoskeletal Biomedical Research Unit, Leeds Teaching Hospitals NHS Trust, Chapel Allerton Hospital, Chapeltown Road, Leeds LS7 4SA, UK

## Abstract

This short article hypothesizes that the major reason for persistent disease despite biologic therapy is the inappropriately late timing of therapy with biologic agents. There is clear evidence to support this hypothesis. This short review will indicate that patients treated at an earlier phase of disease can achieve a clinical remission rate of 70% and a response rate of above 95%.

## 

The majority of studies of rheumatoid arthritis (RA) have shown that when patients with RA are treated with biologics, they achieve a remission rate of only 30% or less. There are many potential reasons why disease activity is incompletely suppressed by biologics. The most commonly used biologics are anti-cytokines, in particular those that block TNF alpha. Therefore, a logical reason for persistent disease activity is either incomplete blockade of the individual cytokine or the fact that multiple cytokines/alternative mechanisms are implicated in the pathogenesis. A pragmatic second reason is that the outcome measures used to measure response include elements other than disease activity/inflammation. Even when inflammation is completely switched off, these outcome measures may not normalize. An example of this is the reduced impact of inflammation-suppressive therapy on outcome measures in later disease, at a time when there is already extensive damage.

This short article, representing a personal view, will examine the hypothesis that a major reason there is persistent disease activity is that anti-cytokine biologics are used inappropriately late in the disease. In patients with late disease, the disease activity score (DAS) reflects damage as well as inflammation and therapy has a large irreversible component. More controversially, the pathogenesis of the disease by this time may have evolved, so that the disease process is less reversible with the blockade of a single cytokine. For both of these reasons, TNF blockade will produce full benefits only when given early in the course of disease.

## The move from inflammation to damage

For patients with RA, biologic treatment is introduced at different stages of disease. These stages can be described in terms of the therapy previously received by the patient. The first stage is the methotrexate (MTX)-naïve patient, the next is the MTX incomplete responder (IR), and the last is TNF inhibitor IR. With movement across the sequence of stages, increasing duration of disease is observed as well as increased complexity of pathogenesis. This latter fact is linked to the partial resistance of the disease to conventional therapy later in the disease course.

The most commonly studied population treated with biologics consists of MTX IR patients with continued disease activity. Such patients fulfill National Institute for Health and Care Excellence guidelines for reimbursement of therapy and worldwide represent the majority of the population treated with biologics. In these patients, the American College of Rheumatology 20% improvement criteria (ACR20), ACR50, and ACR70 responses observed are approximately 60%, 40%, and 20%, respectively [1]. The conventional interpretation of these results is that blockade of TNF produces major suppression of disease in only a minority of patients. The corollary of this is that a very significant part of the disease activity (inflammation) is TNF-independent. Thus, the conventional answer to the question ‘Why is there persistent disease despite biologic therapy?’ is that the disease processes are not completely blocked by inhibition of the cytokine. This short report will focus on some flaws in this analysis by examining responses in very early patients treated from onset with combination MTX and a biologic which inhibits TNF.

## The phenotype of methotrexate incomplete responder patients

It is possible to determine relatively quickly whether a patient is failing MTX. However, the majority of patients classified as MTX IR patients not only will have been taking MTX for a considerable time but also will have substantial disease duration. Patients often have had a diagnosis made several years earlier and therefore will fall into the category of late-disease treatment (reviewed by Aaltonen and colleagues [1]). In addition (as mentioned above), they may well have more complex disease which is less responsive to TNF blockade alone than in MTX-naïve patients.

## Methotrexate-naïve patients

**Figure 1:**
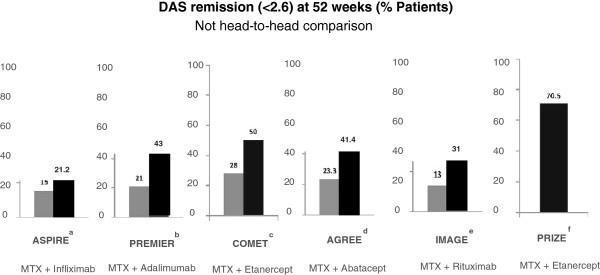
**Remission rates across rheumatoid arthritis studies according to previous therapy. **^a^[4]; ^b^[5]; ^c^[2]; ^d^[6]; ^e^[7]; ^f^[8]. AGREE, Abatacept study to Gauge Remission and joint damage progression in methotrexate-naïve patients with Early Erosive Rheumatoid Arthritis; ASPIRE, Active-Controlled Study of Patients Receiving Infliximab for the Treatment of Early-Onset Rheumatoid Arthritis; COMET, Combination of Methotrexate and Etanercept in Active Early Rheumatoid Arthritis; DAS, disease activity score; IMAGE, A Study to Evaluate Rituximab in Combination With Methotrexate in Methotrexate-Naive Patients With Active Rheumatoid Arthritis; PREMIER, Efficacy and Safety of Adalimumab and Methotrexate (MTX) Versus MTX Monotherapy in Subjects With Early Rheumatoid Arthritis; PRIZE, Productivity and Remission in a Randomized Controlled Trial of Etanercept versus Standard of Care in Early Rheumatoid Arthritis.

In Figure 1, it is clear that MTX-naïve patients achieve better clinical responses to biologics than those reported for either MTX IR or TNF IR. However, even within this population of essentially disease-modifying anti-rheumatic drug-naïve patients, differences in response are seen according to disease/symptom duration. Perhaps the best example of this is the COMET (Combination of Methotrexate and Etanercept in Active Early Rheumatoid Arthritis) study [2], which compared MTX and placebo with MTX and etanercept. The patients were all MTX-naïve and had relatively short disease duration (maximum of 2 years) [2]. The remission rate for the combination was 50%. However, on the basis of the arguments developed in this article, it was decided to re-examine the data according to whether the patients were treated within the first 4 months of their disease. In these patients, remission rates for the combination therapy rose to 70%, whereas those for MTX remained roughly the same at 30% [3]. Furthermore, when patients who had toxicity are excluded, virtually everybody responded.

**Figure 2:**
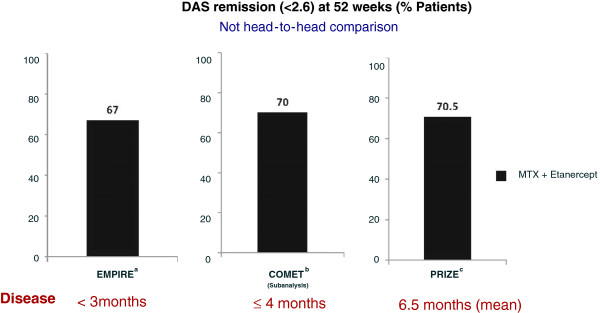
**Remission rates in early rheumatoid arthritis with etanercept/methotrexate. **^a^[9]; ^b^[3]; ^c^[10]. COMET, Combination of Methotrexate and Etanercept in Active Early Rheumatoid Arthritis; DAS, disease activity score; EMPIRE, Etanercept and Methotrexate to Induce Remission in Patients With Newly Diagnosed Inflammatory Arthritis; MTX, methotrexate; PRIZE, Productivity and Remission in a Randomized Controlled Trial of Etanercept versus Standard of Care in Early Rheumatoid Arthritis.

Although this could be seen as a ‘one off’, it has recently been reproduced by other studies: the first is a double-blind randomized controlled study [9] which produced remission rates in the combination MTX and etanercept arm of 68% in patients with very early RA inflammatory arthritis; in the second study, PRIZE (Productivity and Remission in a Randomized Controlled Trial of Etanercept versus Standard of Care in Early Rheumatoid Arthritis), patients in early disease were treated in an open fashion with an intention-to-treat remission rate of 70.5% [10] (Figure 2). The reversibility of inflammation and response to TNF blockade of early disease are clearly seen in the EMPIRE (Etanercept and Methotrexate to Induce Remission in Patients With Newly Diagnosed Inflammatory Arthritis) study, in which clinical remission was observed in 38% after the first injection of etanercept [9]. The findings are supported by previous studies: a randomized controlled study [11] and an open study of infliximab in very early disease treated at presentation had shown over 90% remission rates at week 14 in patients [12]. The former study also demonstrated maintenance of remission at 2 years, 1 year after stopping biologics. Thus, treating produces a magnitude of response not seen or achievable later and in that sense could be viewed as a window of opportunity.

## Summary

When patients are treated at the start of disease with a blockade of TNF and MTX, the following occur:

1. There is an extremely rapid response (38% at 2 weeks) [9].

2. The majority of patients achieve remission, and few have persistent inflammation [3].

3. It does appear that long-term benefit can ensue (with DAS benefits sustained at 8 years) [13].

4. Therefore, the logical time to use anti-cytokine agents to induce remission is at presentation.

Is there an explanation for this? Biomarkers do seem to indicate a change in T-cell phenotype over time (loss of memory T cells), which does correlate with responsiveness to MTX [14]. Furthermore, there do appear to be two types of signaling: one cytokine-dependent, the other largely cytokine-dependent [15]. It is conceivable that the relative importance of these two signaling pathways changes over time.

### Note

This article is part of the collection ‘*Why is there persistent disease despite aggressive therapy of rheumatoid arthritis?*’, edited by Pierre Miossec. Other articles in this series can be found at http://arthritis-research.com/series/residual.

## Abbreviations

ACR20: American College of Rheumatology 20% improvement criteria; DAS: Disease activity score; IR: Incomplete responder; MTX: Methotrexate; RA: Rheumatoid arthritis; TNF: Tumor necrosis factor.

## Competing interests

PE has provided clinical trials and expert advice for AbbVie, BMS, Merck, Novartis, UCB, Pfizer and Roche.
